# Global Routine Vaccination Coverage, 2016

**DOI:** 10.15585/mmwr.mm6645a3

**Published:** 2017-11-17

**Authors:** Leora R. Feldstein, Stephanie Mariat, Marta Gacic-Dobo, Mamadou S. Diallo, Laura M. Conklin, Aaron S. Wallace

**Affiliations:** ^1^Epidemic Intelligence Service, CDC; ^2^Global Immunization Division, CDC; ^3^Department of Immunization, Vaccines and Biologicals, World Health Organization; ^4^Division of Data, Research and Policy, United Nations Children’s Fund.

The *Global Vaccine Action Plan 2011–2020* (GVAP) (*1*), endorsed by the World Health Assembly in 2012, calls on all countries to reach ≥90% national coverage for all vaccines in the country’s routine immunization schedule by 2020. CDC and the World Health Organization (WHO) evaluated the WHO and United Nations Children’s Fund (UNICEF) global vaccination coverage estimates to describe changes in global and regional coverage as of 2016. Global coverage estimates for the third dose of diphtheria and tetanus toxoids and pertussis–containing vaccine (DTP3), the third dose of polio vaccine, and the first dose of measles-containing vaccine (MCV1) have ranged from 84% to 86% since 2010. The dropout rate (the proportion of children who started but did not complete a vaccination series), an indicator of immunization program performance, was estimated to be 5% in 2016 for the 3-dose DTP series, with dropout highest in the African Region (11%) and lowest in the Western Pacific Region (0.4%). During 2010–2016, estimated global coverage with the second MCV dose (MCV2) increased from 21% to 46% by the end of the second year of life and from 39% to 64% when older age groups (3–14 years) were included (*2*). Improvements in national immunization program performance are necessary to reach and sustain high vaccination coverage to increase protection from vaccine-preventable diseases for all persons.

In 1974, the World Health Organization (WHO) established the Expanded Program on Immunization to ensure that all children have access to four routinely recommended vaccines that protect against six diseases (*3*): bacillus Calmette-Guérin vaccine (to protect against tuberculosis), polio, MCV, and DTP. WHO and UNICEF derive national coverage estimates through an annual country-by-country review of all available data, including administrative and survey-based coverage (*4*,*5*). Typically, only doses administered through routine immunization visits are counted.* This report updates a previous report (*6*) and presents global, regional, and national vaccination coverage estimates and trends as of 2016. It also estimates the proportion of surviving infants who did not receive any DTP doses (referred to as ‘left-out’) and the proportion that received 1 or 2 doses of DTP, but did not receive the third dose of DTP (dropped out), using the DTP 3-dose series as an indicator of overall program performance (*3*,*4*).

Globally, 116.5 million children received DTP3 in 2016 compared with 24.2 million in 1980 (Figure), a 300% increase in global DTP3 coverage from 21% to 86%. In 2016, DTP3 coverage ranged from 74% in the WHO African Region to 97% in the Western Pacific Region (Table 1). In all regions, DTP3 coverage has remained stable or increased during 2015–2016. National DTP3 coverage estimates varied from 19% to 99%. Overall, 130 (67%) of 194 countries achieved ≥90% national DTP3 coverage in 2016, an increase from 128 (66%) countries the previous year (*2*). National DTP3 coverage was 80%–89% in 29 countries, 70%–79% in 15 countries and <70% in 20 countries. Among the 19.5 million children worldwide who did not receive 3 DTP doses during the first year of life, 11.8 million (61%) lived in 10 countries: Nigeria (18%), India (16%), Pakistan (7%), Indonesia (6%), Ethiopia (4%), the Democratic Republic of the Congo (3%), Iraq (3%), Angola (2%), Brazil, (1%) and South Africa (1%).

**FIGURE:**
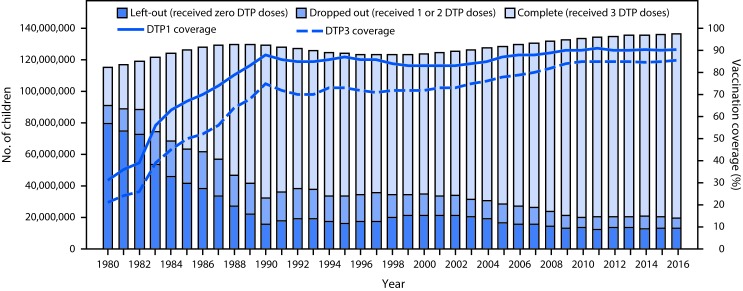
Coverage with the first and third doses of diphtheria and tetanus toxoids and pertussis–containing vaccine (DTP1 and DTP3) and the number of children who were left out (received no DTP doses), dropped out (received 1 or 2 DTP doses), or completed 3 DTP doses — worldwide, 1980–2016

**TABLE 1:** Vaccination coverage, by vaccine and World Health Organization region — worldwide, 2016

Vaccine	No. (%) countries with vaccine in schedule	% Coverage,* by region						
		Global (all regions)	African	Americas	Eastern Mediterranean	European	South-East Asia	Western Pacific
BCG	158 (81)	**88**	81	95	87	91	89	95
HepB BD	84 (43)	**39**	10	66	22	39	34	83
HepB3	185 (95)	**84**	74	89	80	81	88	92
DTP3	194 (100)	**86**	74	91	80	92	88	97
Hib3	191(98)	**70**	74	90	80	77	80	28
Pol3	194(100)	**85**	73	92	80	94	87	95
Rota last	84 (43)	**25**	43	74	23	23	3	2
PCV3	129 (66)	**42**	65	84	48	62	9	14
MCV1	194 (100)	**85**	72	92	77	93	87	96
RCV1	151(78)	**47**	13	92	46	93	15	96
MCV2	160 (82)	**64**	24	54	69	88	75	93

**Abbreviations:** BCG = Bacillus Calmette-Guérin vaccine; DTP3 = third dose of diphtheria and tetanus toxoids and pertussis vaccine; HepB BD = birth dose of hepatitis B vaccine; HepB3 = third dose of hepatitis B vaccine; Hib3 = third dose of *Haemophilus influenzae* type b vaccine; MCV1 = first dose of measles-containing vaccine ; MCV2 = second dose of MCV; PCV3 = 3 doses of pneumococcal conjugate vaccine; Pol3 = third dose of polio vaccine; RCV1 = first dose of rubella-containing vaccine; Rota last = final dose of rotavirus vaccination series.

* BCG coverage based on 158 countries with BCG in the national schedule; coverage for all other vaccines based on 194 countries (global) or all countries in the specified region.

Among approximately 20 million children who did not complete the 3-dose DTP series in 2016, 12.9 million (66%) did not receive any DTP doses, a decrease from 79.4 million in 1980, and 6.6 million (34%) started, but dropped out and did not complete the DTP series (Figure). The largest proportions of infants who were left out were in the WHO African (17%) and Eastern Mediterranean (15%) Regions (Table 2). National DTP1 to DTP3 dropout rates varied from 0% to 55%, with highest dropout in the African Region (11%) and lowest in the Western Pacific Region (0.4%) (Table 2).

**TABLE 2:** Vaccination coverage and dropout proportions for diphtheria and tetanus toxoids and pertussis–containing vaccine and measles-containing vaccine, by World Health Organization (WHO) region — worldwide, 2016

WHO region	DTP1 coverage %	DTP1 left-out* % (No.)	DTP3 coverage %	DTP1 to DTP3 dropout^† ^% (No.)	MCV1 coverage %	MCV1 left-out* % (No.)	MCV2 coverage^§^ %	MCV1 to MCV2 difference^¶^ % (No.)
Global (all regions)	91	9 (12.9M)	86	5 (6.6M)	85	15 (20.8M)	64	21 (29.1M)
African	83	17 (5.9M)	74	11 (3.1M)	72	28 (9.6M)	24	48 (16.2M)
Americas	95	5 (0.7M)	91	4 (0.6M)	92	8 (1.2M)	54	38 (6.0M)
Eastern Mediterranean	85	15 (2.5M)	80	6 (0.8M)	77	24 (3.9M)	69	8 (1.4M)
European	95	5 (0.5M)	92	3 (0.3M)	93	7 (0.8M)	88	5 (0.5M)
South-East Asia	93	7 (2.6M)	88	5 (1.6M)	87	13 (4.5M)	75	12 (4.3M)
Western Pacific	97	3 (0.7M)	97	0.4 (0.08M)	96	4 (0.9M)	93	3 (4.3M)

**Abbreviations:** DTP1 = first dose of diphtheria and tetanus toxoids and pertussis vaccine; DTP3 = third dose of DTP; MCV1 = first dose of measles-containing vaccine; MCV2 = second dose of MCV; M = million.

* Left-out = the proportion of surviving infants who did not receive any doses of the specified vaccine.

^†^ Dropout = those who received 1 or 2 DTP doses but not the third dose of DTP.

^§^ Includes 34 countries that either have not introduced MCV2 or that do not report MCV2 coverage; 65% of these countries are located in the African Region.

^¶^ Difference = children who received MCV1 but not MCV2.

MCV1 coverage in 2016 ranged from 72% in the African Region to 96% in the Western Pacific Region (Table 1) and from 20% to 99% by country. During 2015–2016, MCV1 coverage has remained stable or increased in all regions. Globally, 123 (63%) countries achieved the GVAP 2020 target of ≥90% national MCV1 coverage (*7*).

MCV2 coverage by WHO region varied from 24% (African Region) to 93% (Western Pacific Region), including countries that have not yet introduced MCV2 (Table 1). In four of six WHO regions (African, Region of the Americas, Eastern Mediterranean, and South-East Asia), MCV2 coverage increased in 2016 compared with 2015, because of both an increase in coverage in multiple countries, as well as an increase in the number of countries introducing MCV2. Globally, MCV1 coverage was 85% and MCV2 coverage was 64% in 2016 (estimated dropout = 21%). MCV1 to MCV2 difference was highest in the African Region (48%) and lowest in the Western Pacific Region (3%) (Table 2). This difference represents both lack of MCV2 introduction (34 countries not yet introduced) and differences in program performance. 

Among new and underused vaccines, global coverage increased during 2010–2016 for completed series^†^ of rotavirus vaccine (8% to 25%), pneumococcal conjugate vaccine (PCV) to prevent infections with *Streptococcus pneumoniae* (11% to 42%), rubella vaccine (35% to 47%), *Haemophilus influenzae* type b (Hib) vaccine (42% to 70%), and hepatitis B vaccine (74% to 84%) (Table 1), as a result of improvements in national coverages rates and new country introductions.

## Discussion

Substantial progress in global routine vaccination coverage has been made since 1974, even as the population of surviving infants has increased. As a result, approximately 123 million children, 91% of the global population of surviving infants, received at least 1 dose of DTP vaccine during their first year of life in 2016, and nearly 117 million (86%) completed the DTP series. However, 64 (33%) countries still have not met the GVAP target of ≥90% national DTP3 coverage, and 71 countries (37%) have not reached the 2012–2020 *Global Measles and Rubella Strategic Plan* target of ≥90% national MCV1 coverage (*7*). Moreover, DTP3 and MCV1 coverage rates have remained stagnant since 2010 (*2*). Among the eight countries with DTP3 coverage <50% in 2016 (Central African Republic, Chad, Equatorial Guinea, Nigeria, Somalia, South Sudan, Syria, and Ukraine), nearly all are in conflict or facing serious economic turmoil. Consequently, maintaining coverage with existing vaccines and introducing new vaccines in these countries is particularly challenging.

In light of these challenges, improving initiation of vaccination and completion of the series for all recommended vaccines is an integral step toward improving vaccination coverage globally, particularly in the WHO African, Eastern Mediterranean, and South-East Asia Regions. In these three regions in 2016, 11 million infants did not receive their first dose of DTP vaccine, and 18 million did not receive their first dose of MCV. Six million infants started the DTP series but did not complete it, and 22 million started, but did not complete the MCV series (Table 2). Ensuring that these infants receive the full number of doses of recommended vaccines will be critical to preventing early childhood mortality and morbidity during adolescence and adulthood, and it will provide indirect protection to the whole community.

The findings in this report are subject to at least two limitations. First, numerator and denominator biases might be present because of outdated national census and limited vaccination coverage reporting capabilities at lower administrative levels, which might result in overestimates or underestimates of administrative vaccination coverage. Second, survey data might suffer from recall bias and the data might not be generalizable to the larger population (*4*).

Demographic barriers (minority ethnicity, parents’ lack of education, and low socioeconomic status), populations living in difficult-to-reach areas, programmatic challenges such as vaccine stock-outs, and conflict continue to prevent certain children from receiving the benefits of being fully vaccinated (*8*). At district or country levels, program costs and insufficient political will also contribute to problems with vaccine access and completion of vaccination series (*9*). Strategies to improve vaccine accessibility (i.e., reducing the number of left-out children) might be different from those used to minimize dropout. To improve accessibility, steps are needed to ensure that hard-to-reach populations are identified and that vaccination sessions are made consistently accessible. Program managers need to use effective vaccine management practices to avoid stock-outs, and health workers need to be available and well trained to provide acceptable services to the community (*10*). To minimize dropouts, interventions might include promoting demand for vaccination, particularly in culturally hard-to-reach populations; provider communication to caregivers about the benefits of vaccines and addressing any vaccine safety concerns; reminder or recall strategies to ensure that caregivers return for future vaccinations, and defaulter tracking strategies to identify children who have failed to return for a recommended vaccination. Improving both initiation of vaccination and completion of the series is essential for establishing sustainable national immunization programs and to eliminating preventable diseases and deaths among children.

**Summary**

**What is already known about this topic?**

In 1974, the World Health Organization (WHO) established the Expanded Program on Immunization to ensure that all children have access to routinely recommended vaccines. Since then, global coverage with vaccines to prevent tuberculosis, diphtheria, tetanus, pertussis, poliomyelitis, and measles has increased from <5% to ≥85% and additional vaccines against hepatitis B, *Haemophilus influenzae* type B, *Streptococcus pneumoniae*, rotavirus, and rubella have been included in vaccine recommendations introduced in multiple countries.

**What is added by this report?**

Global coverage with the third dose of diphtheria and tetanus toxoids and pertussis–containing vaccine, the third dose of polio vaccine, and first dose of measles- containing vaccine coverage has remained unchanged at 84%–86% since 2010. Among new or underused vaccines, global coverage increased during 2010–2016 for completed vaccine series against rotavirus (8% to 25%), *Streptococcus pneumoniae* (11% to 42%), rubella (35% to 47%), *Haemophilus influenzae* type B (42% to 70%) and hepatitis B vaccine (74% to 84%). Vaccination coverage varies widely across WHO regions, countries, and districts, with decreased access to vaccination and completion of vaccination series in low-income countries and conflict areas compared with that in other countries.

**What are the implications for public health practice?**

Since 1974, there has been substantial progress in global vaccination; however, in recent years, coverage rates have remained static. This indicates the need to move beyond existing practices to improve access to vaccinations for hard-to-reach populations. Interventions include strengthening caregiver demand for vaccination, provider communication to caregivers about the benefits of vaccines and addressing any vaccine safety concerns; reminder or recall strategies to ensure caregivers return for future vaccinations, and defaulter tracking strategies to identify children who have failed to return for a recommended vaccination.

## Footnotes

* For a given vaccine, the administrative coverage is the number of vaccine doses administered to persons in a specified target group divided by the estimated target population. Doses administered through routine immunization visits are counted, but doses administered through supplemental immunization activities (mass campaigns), usually are not. During vaccination coverage surveys, a representative sample of households is visited, and caregivers of children in a specified target age group (e.g., aged 12–23 months) are interviewed. Dates of vaccination are transcribed from the child's home-based record, recorded based on caregiver recall, or transcribed from health facility records. Survey-based vaccination coverage is calculated as the proportion of persons in a target age group who received a vaccine dose.

† Two rotavirus vaccines are currently licensed; the monovalent vaccine is administered as a 2-dose series, and the pentavalent vaccine is administered as a 3-dose series.
